# Earthquake preparedness of households and its predictors based on health belief model

**DOI:** 10.1186/s12889-020-08814-2

**Published:** 2020-05-08

**Authors:** Masoumeh Rostami-Moez, Mohammad Rabiee-Yeganeh, Mohammadreza Shokouhi, Amin Dosti-Irani, Forouzan Rezapur-Shahkolai

**Affiliations:** 1 Research Center for Health Sciences, Hamadan University of Medical Sciences, Hamadan, Iran; 2 Vice-chancellor for Health, Hamadan University of Medical Sciences, Hamadan, Iran; 3 Chronic Diseases (Home Care) Research Center and School of Nursing & Midwifery, Hamadan University of Medical Sciences, Hamadan, Iran; 4 Department of Epidemiology, School of Health, Hamadan University of Medical Sciences, Hamadan, Iran; 5 Department of Public Health, School of Public Health, Hamadan University of Medical Sciences, Shahid Fahmideh Ave, Hamadan, Iran; 6 Social Determinants of Health Research Center, Hamadan University of Medical Sciences, Hamadan, Iran

**Keywords:** Crises, Hazards, Health promotion, Health education, Natural disasters, Safety

## Abstract

**Background:**

Earthquakes are one of the most destructive natural disasters in which many people are injured, disabled, or died. Iran has only 1 % of the world’s population, but the percentage of its earthquake-related deaths is absolutely higher. Therefore, this study aimed to determine the level of earthquake preparedness of households and its predictors using the Health Belief Model (HBM).

**Methods:**

This observational descriptive and analytical study was conducted on 933 households in Hamadan province, located in the west of Iran, in 2019. Multi-stage cluster random sampling was used for selecting the participants. The inclusion criteria were being at least 18 years old and being able to answer the questions. A questionnaire was used for data collection including earthquake preparedness, awareness of earthquake response, predictors of earthquake preparedness based on the HBM, and demographic information. Analysis of variance, independent t-test, and a linear regression model was used.

**Results:**

The mean age of participants was 38.24 ± 12.85 years. The average score of earthquake preparedness was low (approximately 30%). There was a significant relationship between earthquake preparedness and gender (*P* < 0.001), homeownership (*P* < 0.001), marriage status (*P* < 0.001), education (*P* < 0.001), and previous earthquake experience (*P* < 0.001). Regarding the HBM constructs, perceived benefits (*P* < 0.001), cues to action (*P* < 0.001), and self-efficacy (*P* < 0.001) were significant predictors of earthquake preparedness.

**Conclusions:**

Earthquake preparedness was insufficient. Besides, perceived benefits, cues to action, and self-efficacy were predictors of earthquake preparedness. These predictors can be taken into account, for designing and implementing related future interventions.

## Background

Earthquakes are one of the most dangerous natural hazards that occur suddenly and uncontrollably. They cause physical, psychological, and social damages in human societies [1]. Over the past two decades, 800 million people have been injured by natural disasters. Besides, natural disasters have caused 42 million deaths in the world [2]. Iran is always at risk of earthquakes due to its geographical location on the Alpine-Himalayan orogenic belt [3, 4]. More than 70% of the major cities in Iran are vulnerable to substantial damages. The earthquakes of recent decades have not only caused the deaths of thousands but also have caused massive economic damage and destroyed many cities and villages in the world [5, 6]. Iran has only 1 % of the world’s population, but the percentage of its earthquake-related deaths is absolutely higher [7]. The disaster management cycle has four phases including mitigation, preparedness, response, and recovery. Preparedness is the most important phase in the disaster management cycle. Previous research in Iran has shown that the role of people as the most important and largest group has often been neglected in disaster preparedness program planning [8].

The Health Belief Model (HBM) describes the decision-making process that individuals use to adopt healthy behavior. It can be an effective framework for developing health promotion strategies [9]. Theoretically, in the HBM, perceived susceptibility, perceived severity, perceived benefits, perceived barriers, cues to action, and self-efficacy (the beliefs of individuals in their ability to prepare for disaster) predict behavior [1, 9, 10].

There are some studies on earthquake preparedness that have assessed the readiness of individuals based on their knowledge and skills [11–15]. Some studies have also considered structural and non-structural safety in some cities [16] and some studies have investigated students’ readiness [17, 18]. There are a few studies that have used behavioral change models in the disaster area [5]. The Haraoka and Inal used the Health Belief Model to develop a questionnaire for earthquake preparedness [1, 11].

Previous studies in Iran showed that most households did not have enough readiness and had a relatively high vulnerability to possible earthquake hazards [19, 20]. Also, one study showed that improving the socio-economic status was correlated with improving the attitude of people about disaster preparedness [13]. In DeYoung et al.ʼs study, earthquake readiness was positively correlated with risk perception, self-efficacy, and trust in information about hazards through media [21].

To the best of the authors’ knowledge, this is the first study in Iran that examines earthquake preparedness of households, using a behavior change model. Considering the importance of earthquake preparedness of households, this study aims to asses the level of earthquake preparedness of households and its predictors based on HBM.

## Methods

### Study design and participants

This observational descriptive and analytical study was carried out in all counties of Hamadan province, located in the west of Iran, in 2019. These counties includes Hamadan (the capital of Hamadan province), Malayer, Tuyserkan, Nahavand, Razan, Bahar, Kabudarahang, Asadabad, and Famenin. Based on the previous study [19], the estimated sample size was 600 households. Cluster sampling was used for this study and we used the design effect of 1.5 plus 10% attrition. Subsequently, the final sample size was calculated at 1000 households. The data were collected from February to July 2019. From each county, a university graduate person was recruited and trained for data collection. The supervision and training were done by the first author. The verbal informed consent was obtained from all participants before the data gathering. The participants were first provided a description of the study and they were informed that the participation in the study was voluntary, and all study data were anonymous and confidential. Then, if they gave verbal informed consent, they would participate in the study and fill out the anonymous questionnaires. A person aged 18 or above was randomly selected from each household and answered the questions. For illiterate people, questionnaires were filled out through interviewing them. The inclusion criteria were being at least 18 years old and being able to answer the questions. The exclusion criteria were an incomplete questionnaire.

Participants have been selected by multi-stage cluster random sampling. First, stratified sampling was used for each county based on its urban and rural populations. Then, in urban and rural areas, a list of urban or rural health centers was listed and one health center was randomly selected in each county. After that, from the list of all households covered by the selected health center, one household was selected by simple random sampling and sampling started taking the clockwise direction of the selected household and continued until the required sample was collected. For selecting the sample of the urban population of Hamadan County, we selected one health center from each district by simple random sampling (in Hamadan city, there are four districts). In the next stage, from the list of covered households, one household was randomly selected and the sampling was started taking the clockwise direction until the required sample in each district was collected.

### Measurements

The questionnaire used for data collection comprises four domains including 1) demographics, 2) earthquake preparedness 3) awareness on earthquake response, and 4) predictor of earthquake preparedness based on the HBM. Earthquake preparedness was response variable.

1. Demographics included age, sex, occupation, education, economic status, family size, number of individuals over 60 years old and under 16, earthquake experience, homeownership, marital status, and having a person with a disease that needs medication at their home.
2. We measured earthquake preparedness by an earthquake preparedness checklist [22]. This checklist was developed and validated by Spittal et al., in 2006. It consists of 23 questions with yes or no answers. The questions are about: having a working torch (flashlight), a first aid kit, a working battery radio, a working fire extinguisher, etc. [22]. We adapted this checklist by adding two items according to the context of the study. These two questions were: 1) do you know the necessary contact numbers such as fire station, police, and emergency so that you will be able to call them if needed?; 2) are you familiar with the phrase, “Drop, Cover, and Hold”? Also, we adapted it with some minor changes. We added “have learned first aid” to “have purchased first aid kit” statement. We added “and extra cloths and blankets” at the end of” put aside extra plastic bags and toilet paper for use as an emergency toilet” statement. We replaced “roof” with “my way” in “ensuring that the roof will probably not collapse in an earthquake. We added some examples to “take some steps at work” statement such as attending an earthquake preparedness class and having fire insurance. The content validity of the Persian checklist was tested by 10 experts. We calculated CVI and CVR equal to 0.92 and 0.95, respectively. Also, the face validity and reliability of this checklist were examined in a pilot study on 40 adults. According to their recommendations, minor revisions were made to increase the transparency and understandability of the statements. Likewise, the reliability of this checklist was measured by internal consistency (Chronbach α = 0.858). The total score of this checklist was ranging from 0 to 25 and the higher score reflects more preparedness.
3. The awareness on earthquake response questionnaire included seven questions with true/false answers (In an earthquake: you should get down close to the ground; you should get under a big piece of furniture such as a desk or other covers; you should hold on to a firm object until the end of the shaking; you should stand in a doorway; If you are indoors during an earthquake, you must exit the building; If you are in bed during an earthquake, you should stay there and cover your head with a pillow; next to pillars of buildings and interior wall corners are the safe areas). One point was given for each correct answer. Therefore, the total score of this domain was seven points.
4. The adapted questionnaire of earthquake preparedness based on the HBM was used. The original questionnaire has been established and validated by Inal et al. [1] in Turkey. The forward and backward translation method was used for translating the original questionnaire. According to the experts’ opinions, some minor changes were made to adapt the items of the questionnaire for the study population in the present study. Thereby, three questions were added to the questions of the cues to action (Radio and TV encourage me to prepare for disasters, I usually seek information about disaster preparedness from Radio and TV, and I usually obtain information about disaster preparedness from health providers). Besides, one question was added to the questions of perceived benefits (preparedness for disaster will reduce financial losses and injuries). Then, the content validity of the questionnaire was assessed by a panel of experts including 10 Health specialists in the field of health in disasters, health education, health promotion, and safety promotion (CVR = 0.92 & CVI = 0.85). Next, the face validity and reliability of the questionnaire were measured in a pilot study on 40 people over 18 years old. The reliability was calculated by using internal consistency. One question from the perceived severity (emergency and the experience of disasters does not change my life) and one question from self-efficacy (I cannot create an emergency plan with my neighbors) was excluded based on the results of Cronbach’s alpha. In Iran, neighbors don’t share their plans; therefore, it was logical to exclude these items. Finally, the questionnaire consisted of 33 questions, including perceived severity (2 questions, α = 0.709), perceived susceptibility (6 questions, α = 0.664), perceived benefits (4 questions, α = 0.758), perceived barriers (6 questions, α = 0.822), self-efficacy (7 questions, α = 0.677), cues to action (8 questions, α = 0.683), and total questions (33 questions, α = 0.809). All of the items were assessed by a 5-point Likert scale ranging from ‘completely disagree’ (one point) to ‘completely agree’ (5 points). Some items were scored reversely.

### Statistical analysis

We used the analysis of variance (ANOVA) and independent t-test to determine the relationship between variables. Besides, the multivariate linear regression model was used to determine the predictors of household earthquake preparedness. The Stata 14.2 software was used to analyze the data.

## Results

In this study, 933 questionnaires were analyzed (response rate: 93.3%). The mean age of participants was 38.24 ± 12.85 years. Besides, 228 (24.44%) participants were male and 656 (70.31%) were female. About 80% of the participants did not have an academic education and had a diploma degree or less than a diploma degree. Also, 573 (61.41%) participants were homeowners (Table 1).

**Table 1:** Basic and demographic characteristics of participants of earthquake preparedness study

Variables		Mean	SD
Age		38.24	12.85
Family size		3.74	1.29
Residential years		13.09	12.58
Variables		Number	Percent
Sex	Man	228	24.44
	Woman	656	70.31
Education	Uneducated	86	9.22
	Primary	216	23.15
	Secondary	187	20.04
	High school	77	8.25
	Diploma	179	19.19
	University	186	19.94
Economic statue	Very good	22	2.36
	Good	43	4.61
	Moderate	387	41.48
	Poor	243	26.05
	Very poor	205	21.97
Owner of the house	Yes	573	61.41
	No	313	33.55
Marriage statue	Married	812	87.03
	Unmarried	121	12.07
Earthquake experience	Yes	744	79.74
	No	136	136
Damaging earthquake experience	Yes	66	7.07
	No	822	88.10
Does anyone over 60 years old live at your home?	Yes	253	27.12
	No	680	72.88
Does anyone under 16 years old live at your home?	Yes	597	63.99
	No	336	36.01
Does anyone live with a disease that needs medication at your home?	Yes	194	20.79
	No	736	78.89

The earthquake preparedness of the participants was low. The household preparedness score was 7.5 out of 25. In other words, the average earthquake preparedness of households was approximately 30%. Besides, the self-efficacy score was 60.79 ± 0.55 and the score of cues to action was 66.57 ± 0.45 (Table 2).

**Table 2:** The mean scores (in percentage) of earthquake preparedness, constructs of Health Belief Model, and earthquake performance awareness of participants

Variables	Mean (SD)	95%CI
Earthquake Preparedness	30.03 (0.65)	28.31–31.31
Perceived susceptibility	68.67 (0.55)	67.59–69.75
Perceived severity	73.16 (0.77)	71.65–74.61
Perceived benefits	77.51 (0.69)	76.15–78.87
Perceived barriers	50.55 (0.52)	49.53–51.57
Cues to action	66.57 (0.45)	46.46–65.67
Self-Efficacy	60.79 (0.55)	59.71–61.87
Earthquake performance awareness	57.61 (0.71)	56.21–59.02

The participants’ preparedness for the earthquake had a significant relationship with gender (*P* < 0.001), homeownership (*P* < 0.001), marital status (*P* < 0.001), and previous experience of a destructive earthquake (*P* < 0.001). Also, the mean score of earthquake preparedness was higher in those who reported moderate or good economic status. The mean difference was statistically significant by the Scheffe test (*P* < 0.001). Furthermore, the one-way ANOVA/Scheffe’s test showed that there was a significant difference between illiterate people and those who had either university education or diploma degree and similarly, a significant difference in earthquake preparedness was observed between primary education and those who had either academic education or diploma degree (*P* < 0.001) (Table 3).

**Table 3:** The relationship between earthquake preparedness and demographic variables of participants by Independent T-Test and Analysis of Variance

Variables		Mean	Standard error	*P*-value
Sex	female	30.560	0.781	0.001>
	Male	29.004	1.281	
Homeowner	Yes	31.05	0.827	0.001>
	No	27.961	1.084	
Marriage statue	Married	30.182	0.693	0.001>
	Unmarried	28.740	1.903	
Earthquake experience	Yes	30.951	0.715	0.001 >
	No	25.825	1.686	
Destructive earthquake experience	Yes	35.151	2.461	0.001 >
	No	29.888	0.685	
Age	20>	30.511	3.252	0.216
	21–30	30.971	1.246	
	31–40	31.265	1.103	
	41–50	29.541	1.446	
	50<	26.834	1.705	
Educational level	illiterate	25.116	1.194	0.001 >
	Elementary	27.574	1.300	
	Secondary school	27.336	1.396	
	High school	29.368	1.172	
	Diploma	33.810	1.509	
	Academic	34.774	1.436	
Economic statue	Very poor	26.595	1.224	0.001>
	Poor	25.272	1.239	
	Moderate	33.385	1.008	
	Good	40.093	3.212	
	Very good	32.909	4.700	
Residential type	With yard	30.668	0.813	
	Apartment	32.699	1.568	0.058
	Leased	26.173	1.473	
	Mortgage	31.076	5.586	
	Governmental	31	8.22	

The crude regression analysis showed that all constructs of the HBM except perceived severity were significant predictors of earthquake preparedness (P < 0.001) but after using stepwise regression, only perceived benefits (*P* < 0.006), cues to action (*P* < 0.001), and self-efficacy (*P* < 0.001), significantly predicted the earthquake preparedness (Table 4).

**Table 4:** The relationship between earthquake preparedness and study variables, using Stepwise Linear Regression

Variables	Regression coefficient	*p*-value	95%CI	
			Lower	upper
Perceived susceptibility	0.005	0.931	-0.106	0.116
Perceived severity	-0.020	0.554	-0.087	0.046
Perceived benefits	-0.125	0.006	-0.214	-0.036
Perceived barriers	0.060	0.267	-0.047	0.169
Cues to action	0.318	0.001	0.197	0.439
Self-efficacy	0.253	0.001	0.137	0.369
Awareness	0.048	0.146	-0.016	0.113
Education	1.793	0.001	0.854	2.733
Earthquake experience	2.561	0.198	-1.337	6.459
Economic statue	1.580	0.053	-0.22	3.182
Residential type	-2.467	0.004	-4.151	-0.783
Household size	-1.365	0.016	-2.479	-0.251
Destructive earthquake experience	7.855	0.004	2.466	12.843

## Discussion

In this study, we determined the level of earthquake preparedness of households and its predictors based on HBM. The earthquake preparedness of the participants was low. The participants’ preparedness for the earthquake had a significant relationship with homeownership, education, and previous experience of a destructive earthquake. Also, perceived benefits, cues to action, and self-efficacy significantly predicted the earthquake preparedness.

Despite the strong emphasis on earthquake preparedness to prevent its damaging effects, the findings of this study showed that most people had low preparedness for earthquakes which is similar to the findings of previous studies [18, 23–25]. This can be very dangerous in areas that are vulnerable to earthquakes. Earthquake preparedness is related to the previous experience of destructive earthquakes and their damaging consequences. Households that had previously experienced destructive earthquakes were more prepared than those who had not previously experienced this event, which is similar to previous finding [26, 27]. People who live in earthquakes zones and understand the potential losses from earthquakes are more likely to be prepared in comparison to people living in other areas [18]. This could be due to recalling previous injuries as well as the fear of recurrence of similar injuries in future earthquakes. This goes back to the culture of societies that their members don’t believe that they are at risk of the occurrence of hazards and their consequences until they experience these hazards. Regarding the high frequency of earthquakes in the Hamadan province, most of the participants in this study had previous earthquake experience but they were not prepared for earthquakes. Perhaps this is because most of the recent earthquakes in Hamadan did not result in deaths and as a result, these households do not take the risk of earthquakes seriously and do not find it essential to hold earthquake preparedness [28].

Besides, education was significantly correlated with households’ earthquake preparedness, which is similar to the results of the studies by Russell et al. and Ghadiri & Nasabi [29, 30]. One explanation can be that people with higher education are more knowledgeable, more aware of earthquakes danger, and more inclined to acquire new skills [28, 31].

In this study, we found that the preparedness of participants has a significant relationship with homeownership. Two previous studies showed homeowners were more prepared for earthquakes than renters [32, 33], whereas a study in Ethiopia in 2014 showed that homeownership had no relationship with disaster preparedness [28]. One of the explanations is that owners can make the necessary changes despite preparedness costs due to place attachment, but more studies are required to confirm the role of homeownership.

We adjusted for multiple possibly confounding factors in our analysis. After adjusting the model, perceived benefit, cues to action, and self-efficacy had significant predictors of earthquake preparedness. It is more possible that people’s earthquake preparedness increases when they are aware of the benefits of earthquake preparedness. Furthermore, people with high self-efficacy feel they can prepare for earthquakes [34]. On the other hand, people may find the earthquake hazardous but if they feel enough confident to reduce damages of earthquakes, they will engage in preparedness. If people perceive the benefits of a healthy behavior higher than the barriers of it, they will engage in that healthy behavior. Therefore, people may perceive earthquakes as a high threat but it can be expected that higher perceived benefits and self-efficacy among them result in higher preparedness. One possible explanation is that the perceived benefits motivate people to perform a specific behavior and adopt an action [10]. Besides, the significant association of self-efficacy with preparedness at the household level for earthquakes could be explained by the positive and strong association of cues to actions with earthquake preparedness at the household level. Self-efficacy can be improved by observational learning, role modeling, and encouragement. Self-efficacy affects one’s efforts to change risk behavior and causes the continuation of one’s safe behavior despite obstacles that may decrease motivation [10]. Moreover, cues to action associated with earthquake preparedness [1]. Cues to action mention to influences of the social environment such as family, friends, and mass media. Mass media can play a vital role in educating the public about earthquake preparedness.

This study has several limitations. Firstly, using a self-reporting approach for data gathering, and secondly, due to the low number of relevant studies on earthquake preparedness based on behavioral change models, it was less possible to compare different studies with the findings of this study. Third, it should be noted that the results of this study can be generalized in the study population and setting, but for other settings it should be done with caution. Despite these limitations, this study had some strengths, we use a theoretical framework for identifying factors that influence earthquake preparedness with a large sample size. Also, the findings of this study are useful for emergency service providers, health authorities, and policymakers in designing and implementing earthquake preparedness programs. This research is also useful for researchers as it can be used as a basis for future researches. It is recommended to design and implement interventions to improve household preparedness for an earthquake based on self-efficacy, perceived benefits, and cues to action.

## Conclusion

Households’ earthquake preparedness was insufficient and low. Controlling the damaging consequences of earthquakes is related to the preparedness for earthquakes and can prevent its devastating effects. Perceived benefits, cues to action, and self-efficacy had a significant relationship with earthquake preparedness. The possibility of people being more prepared is increased when they are aware of and understand properly the benefits of being prepared for earthquakes and other disasters. People with high self-efficacy also feel more empowered for taking better care of themselves and their families during disasters. Cues to action would also encourage earthquake preparedness. Since health centers and TV and radio programs were the primary sources of learning about earthquakes for the people, it is recommended that broadcasting provides related programs and educates people about earthquake preparedness. The predictors that were assessed in this study can be taken into account for designing and implementing proper interventions in this field.

## Data Availability

The analyzed datasets during this study are available from the corresponding author on reasonable request.

## Abbreviations

HBM: Health Belief Model
CI: Confidence Interval
ANOVA: Analysis of Variance
CVR: Content Validity Ratio
CVI: Content Validity Index
